# Prognostic value of tumor immune cell infiltration patterns in colon adenocarcinoma based on systematic bioinformatics analysis

**DOI:** 10.1186/s12935-021-02048-x

**Published:** 2021-07-03

**Authors:** Hao Xu, Qianhui Xu, Lu Yin

**Affiliations:** 1 Department of Hepatobiliary and Pancreatic Surgery, The Second Affiliated Hospital, School of Medicine, Zhejiang University, Hangzhou, Zhejiang China; 2 Department of Pathology, Affiliated Hangzhou First People’s Hospital, Zhejiang University School of Medicine, Hangzhou, Zhejiang China

**Keywords:** Colon adenocarcinoma, Tumor microenvironment, Immune cell infiltration, Prognosis, Immunotherapy

## Abstract

**Background:**

Although immunotherapy for colon cancer has made promising progress, only a few patients currently benefit from it. A recent study revealed that infiltrating immune cells are highly relevant to tumor prognosis and influence the expression of immune-related genes. However, the characterization of immune cell infiltration (ICI) has not yet been comprehensively analyzed and quantified in colon adenocarcinoma (COAD).

**Methods:**

The multiomic data of COAD samples were downloaded from TCGA. ESTIMATE algorithm, ssGSEA method and CIBERSORT analysis were conducted to estimate the subpopulations of infiltrating immune cells. COAD subtypes based on ICI pattern were identified by consensus clustering then principal-component analysis was performed to obtain ICI scores to quantify the ICI patterns in individual tumors. Kaplan–Meier analysis was employed to validate prognostic value. Gene set enrichment analysis (GSEA) was applied for functional annotation. Finally, the mutation data was analyzed by employing “maftools” package.

**Results:**

Three bioinformatics algorithms were used to evaluate the ICI patterns from 538 patients with COAD. Two ICI subtypes were determined using consensus clustering, and the ICI score was constructed by performing principal component analysis. Our findings showed that a higher ICI score often indicated a more advanced tumor and worse prognosis. The high-ICI score subgroup had a higher stromal score and more M0 macrophages but fewer plasma cells and decreased CD8 T cell infiltration. In addition, patients with high ICI scores had significantly higher expression levels of HAVCR2 and PCDC1LG2. Real-time polymerase chain reaction (PCR) was conducted to determine the prognostic significances of ICI-related genes.

**Conclusions:**

In conclusion, ICI score may be considered as an original and useful indicator for independent prognostic prediction and individual immune-related therapy.

**Supplementary Information:**

The online version contains supplementary material available at 10.1186/s12935-021-02048-x.

## Introduction

Colon adenocarcinoma (COAD), a type of colorectal cancer (CRC), is the third most common cancer and the third leading cause of cancer-related deaths in both men and women [1]. With the widespread use of early diagnosis technology (such as colonoscopy), the incidence of COAD has been decreasing since the 2000s [1]. However, due to the lack of obvious clinical symptoms of early colon cancer and frequent metastasis, a large number of patients are already in the advanced stage of colon cancer prior to arriving at the hospital, which poses great challenges for treatment. Thus, there is an urgent need to alter the current therapeutic strategy in order to improve patient prognosis.

At present, colon cancer is primarily treated with surgery, radiotherapy, and chemotherapy. Recently, immunotherapy, especially immune checkpoint blockade (ICB), has emerged as an original method that exerts surprising therapeutic effects in various types of cancers, such as melanoma as well as renal and lung carcinoma [2]. Based on the distinct DNA proofreading and repair mechanisms, COAD can be classified into two molecular subsets. Patients with mismatch repair deficiency (dMMR) and DNA polymerase epsilon mutations are usually accompanied by a higher degree of T cell infiltration and response to immune checkpoint inhibitors, which indicates a better clinical prognosis [3]. However, only a limited number of patients with a high mutation burden can benefit from immune checkpoint inhibitors. Hence, we aimed to identify new therapeutic markers to determine the subgroups with immunotherapy sensitivity.

The tumor microenvironment (TME) is a vastly complicated cellular network composed of tumor cells, stromal cells, soluble factors, signal molecules, and extracellular matrix components [4]. Recent studies have revealed that the interactions between cancer cells and their surrounding TME could affect recruitment and activation of immune cells, tumor angiogenesis, and extracellular matrix remodeling, which determine tumor progression [5, 6]. As an important part of TME, infiltrating immune cells, such as macrophages and lymphocytes, are considered to be highly relevant to tumor prognosis and influence the expression of immune-related genes in CRC [7]. Therefore, the patterns of immune cell infiltration (ICI) may possess potential prognostic value and be used to guide immunotherapy.

In this study, 538 COAD patients were clustered into 2 subgroups based on their ICI patterns. Subsequently, we used three algorithms, named “CIBERSORT,” “ESTIMATE,” and “ssGSEA,” to evaluate the gene expression information of COAD and develop a systematic landscape of tumor ICI. Additionally, we calculated the ICI scores, which could uncover the ICI patterns, for precise prognostic prediction and immunotherapeutic guidance. Finally, prognostic roles of the target gene were validated in vitro.

## Materials and methods

### COAD data and sample collection

A total 538 COAD samples, including RNA sequencing transcriptomic and clinical data, were obtained from The Cancer Genome Atlas (TCGA; http://cancergenome.nih.gov) and Gene Expression Omnibus (GEO; GenBank: GSE29623; https://www.ncbi.nlm.nih.gov/geo) databases. The expression patterns of TCGA-COAD samples were transformed from FPKMs (Fragments Per Kilo-downloaded Million) into TPMs (Transcripts Per Kilobase Million). The “ComBat” algorithm was also used to eliminate the possibility of batch effects caused by non-biotech bias among different datasets [8]. The specific clinicopathological data from TCGA-COAD and GSE29623 were listed in Additional file 1: Tables S5 and S6, respectively.

### ICI patterns in the immune-related TME

Using the R software package “CIBERSORT” (http://cibersort.stanford.edu), the gene expression levels of the TCGA-COAD and GSE29623 combination cohort were evaluated to draw the ICI matrix [9]. Subsequently, the “ESTIMATE” algorithm was performed to calculate the stromal score, immune score, and estimate score for each COAD sample Yu, Wang [10]. In addition, the R package “GSEABase” was executed for 29 immune-related signatures to further reveal the prospects of immune-related responses.

### Consensus clustering for tumor ICI

In order to further stratify the COAD samples into different subgroups, the unsupervised clustering “Pam” method, which is based on the Euclidean and Ward linkage methods, was conducted using the “ConsensuClusterPlus” R software package and repeated as many as 1000 times to ensure classification stability in this analysis.

### Differentially expressed genes (DEGs) of ICI clusters and enrichment analysis

According to ICI patterns, COAD patients from the TCGA-COAD and GSE29623 datasets were divided into different ICI clusters. To identify the genes related to ICI patterns, DEG analysis between ICI subgroups was performed using the R software package “limma.” The significant cutoff thresholds for determining DEGs were P < 0.05 after adjustment and absolute fold change > 1.5. To further clarify the biological functions of the DEGs, Gene Ontology enrichment analysis was performed to annotate the genes that were differentially expressed between different subgroups.

### Definition and generation of ICI scores

First, patients from the TCGA and GEO combination cohort were classified according to the DEG values using unsupervised clustering. The DEG values, which were positively correlated and negatively correlated with cluster signatures, were named as ICI gene signatures A and B, respectively. Next, the Boruta algorithm was used for dimensionality reduction of the ICI gene signatures A and B [11]. Subsequently, we extracted principal component 1 and performed principal component analysis (PCA) to generate the signature score. Finally, the ICI score of every COAD patient was defined using a method similar to the gene expression grade index [12] (ICI score = ∑PC1_A_—∑PC1_B_).

### Correlation between ICI score and immunotherapy

To explore the immune infiltrating landscape of the TME in the high and low ICI score subgroups, we performed CIBERSORT, ESTIMATE, and GSEABase assays as described above. In addition, to evaluate the potential value of the ICI score for immunotherapy, we examined the expression of 12 immune-related genes (CD274 [also known as PD-L1], CTLA4, HAVCR2 [also known as TIM3], IDO1, PDCD1 [also known as PD-1], PDCD1LG2 [also known as PD-L2], CD8A, CXCL10, CXCL9, GZMA, GZMB, IFNG, PRF1, TBX2, and TNF). Furthermore, owing to the excellent clinical outcome of ICB therapy, we analyzed the correlation between the ICI score and six crucial ICB immune genes (CD274, CTLA4, HAVCR2, IDO1, PDCD1, and PDCD1LG2).

### Epigenetic mutation data processing

Somatic mutational information was systematically collected and downloaded from the TCGA-COAD dataset. Subsequently, we calculated the COAD mutational burden by counting the total number of non-synonymous mutations. Finally, we used the R software package “maftool” to identify the driver genes of COAD and listed the top 20 driver genes with the highest mutational frequencies [13].

### Patient data and tissue specimens

COAD tissues together with adjacent colon tissues were acquired from patients underwent surgical resection between October 2014 and December 2014. Corresponding adjacent tissues were harvested 3 cm from the edges of the tumor lesion. Tissue specimens were immediately put into liquid nitrogen post-operation. The tissues were then stored into a − 80℃ refrigerator for total RNA extraction. To control the potential confounding factors, all patients were diagnosed with COAD by histopathological examination, while the patients received chemotherapy or radiotherapy were excluded in the study. All participants have signed written informed consent form. In this study, three pairs of tumor and adjacent tissues were employed for further analysis. The specific clinicopathological data was listed in Additional file 1: Table S8.

### Quantitative real-time PCR (qRT-PCR) analysis

Total RNA was isolated from tumors samples and normal tissues using Trizol reagent (Invitrogen, Carlsbad, CA, USA) according to manufacturer’s instructions, and RNA concentration and purity were analyzed in triplicate by Nanodrop 2000 spectrophotometer (Thermo Scientific Inc., Waltham, MA, 93 USA). After that, the total RNA was reverse transcribed to cDNA using a cDNA Reverse Transcription Kit (Vazyme, Nanjing, China). To determine the expression level of CLCA1 and LTLN1, cDNA sample was tested with quantitative real-time polymerase chain reaction (RT-PCR), using (Roche, Basel, Switzerland). All samples were tested at least three times. The expression of glyceraldehyde-3-phosphate dehydrogenase (GAPDH) was used as an endogenous control, and relative expression of CLCA1 and LTLN1 was calculated by comparative Ct method formula 2-ΔΔCt. The sequences of all PCR primers used were as follows (5′–3′): CLCA1: ACAGGGACACACTCGCCAAA (forward), GTCTTCCCCATCCGTCAGCA (reverse); LTLN1: CGGATGTAACACTGAGCACCACTG (forward), TTATCTCACGGCTGCTGCTGTAAC (reverse); GAPDH: GGAGCGAGATCCCTCCAAAAT (forward), GGCTGTTGTCATACTTCTCATGG (reverse).

### Ethics approval and consent to participate

This retrospective study was conducted according to the ethical standards of the responsible committee on human experimentation (institutional and national) and with the Helsinki Declaration of 1975, as revised in 2000. All participants have signed written informed consent form. Ethical approval (No. 2015–425) was obtained from the Ethics Committee of the First Affiliated Hospital, School of Medicine, Zhejiang University.

### Statistical analysis

The Wilcoxon test was used to compare two groups, and the Kruskal–Wallis test was conducted to compare three or more groups. Survival curves were depicted using the Kaplan–Meier plotter (log-rank test). X-tile software was employed to classify COAD patients into different subgroups while decreasing the computational batch effect [14]. Additionally, the chi-square test and Spearman analysis were carried out to evaluate the correlation and calculate the correlation coefficient, respectively. All statistical data were analyzed using R software (version 4.0.3; The R Foundation, Vienna, Austria). Statistical significance was set at P < 0.05.

## Results

### ICI landscape in the TME of COAD

**Fig. 1:**
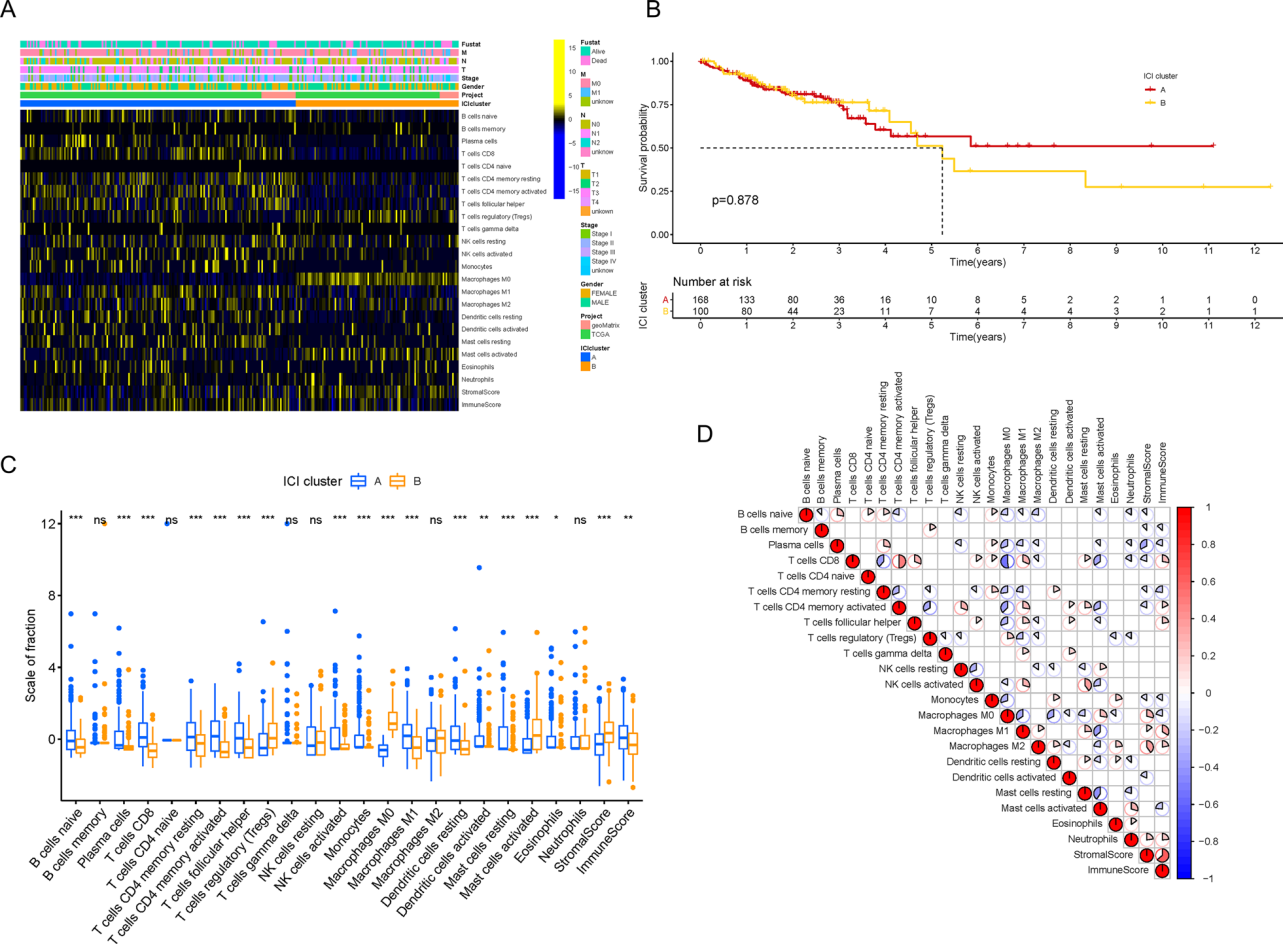
The Landscape of Immune Cell Infiltration in the TME of COAD. **A** Unsupervised clustering of tumor infiltrating immune cells in COAD patients. The rows represent tumor infiltrating immune cells, and the columns represent samples. **B** Kaplan–Meier plotter for COAD patients in two ICI clusters. **C** The proportion of tumor infiltrating immune cells (also including stromal scores and immune scores) in two ICI clusters. **D** Intrinsic interaction of tumor infiltrating immune cells, immune scores and stromal scores

**Fig. 2:**
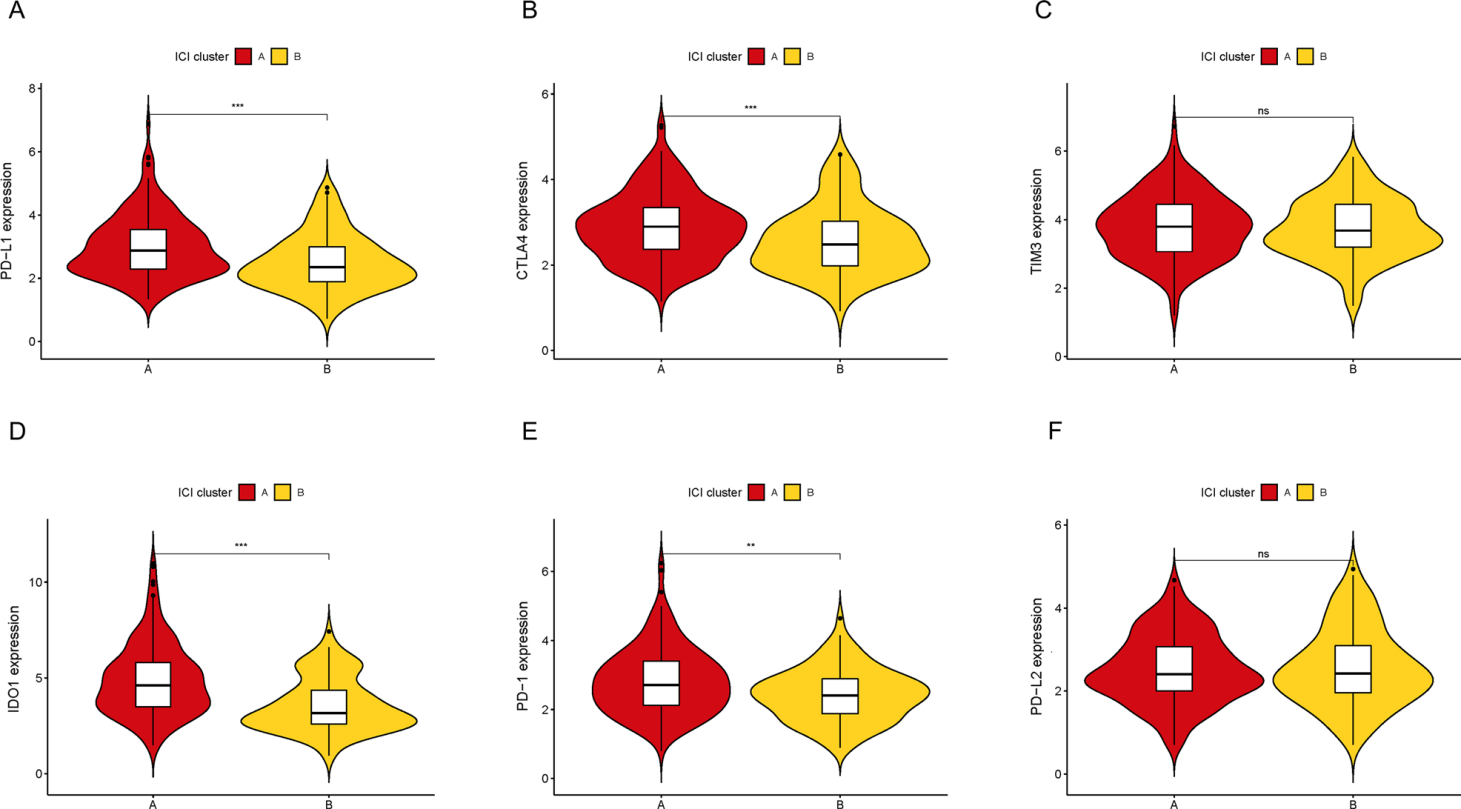
Comparison of ICB genes between two ICI clusters. The expression levels of PD-L1 (**A)**, CTLA4 (**B**), TIM3 (**C**), IDO1 (**D**), PD1 (**E**) and PD-L2 (**F**) of COAD patients from different ICI clusters

To assess the activity and enrichment levels of the inflammatory cell subpopulation in the TME of CRC, we implemented the ESTIMATE and CIBERSORT algorithms. Based on the ICI patterns of 538 CRC samples from the TCGA-COAD and GSE29623 datasets, we clustered the CRC patients into different subgroups using the R software package “ConsensusClusterPlus.” According to the similarity shown in the ICI profiles, the consensus matrix had the best clustering stability when k = 2, and the cumulative distribution function value, which is considered as an indicator of outstanding clustering, tended to increase (Additional file 2: Figure S1A–F). Consequently, we divided these CRC cases into two ICI clusters and depicted the relationship between ICI patterns and clinical phenotypes in the comprehensive heatmap (Fig. 1A). Although there was no significant difference in overall survival (OS) time as shown by the Kaplan–Meier plotter (Fig. 1B; P = 0.878), we observed that the OS of ICI cluster A was higher than that of ICI cluster B after 5 years. Subsequently, in order to uncover the intrinsic biological distinction, we compared the immune cell composition of the two ICI clusters in the TME and visualized the universal ICI landscape using the correlation coefficient heatmap (Fig. 1C and D). We found that ICI cluster A had greater proportions of naïve B cells, plasma cells, CD8 T cells, resting or activated memory CD4 T cells, follicular helper T cells, activated NK cells, monocytes, M1 macrophages, and resting or activated dendritic cells as well as a higher immune score. On the other hand, ICI cluster B possessed markedly increasing densities of regulatory T cells, M0 macrophages, and activated mast cells as well as a higher stromal score. We also estimated the expression levels of several ICB genes between the two ICI clusters, such as PD-L1, CTLA4, TIM3, IDO1, PD-1, and PD-L2 (Fig. 2A–F). We observed that all six genes of ICI cluster A, except TIM3 and PD-L2, were expressed at higher levels than those of ICI cluster B.

### Determined immune gene subsets

**Fig. 3:**
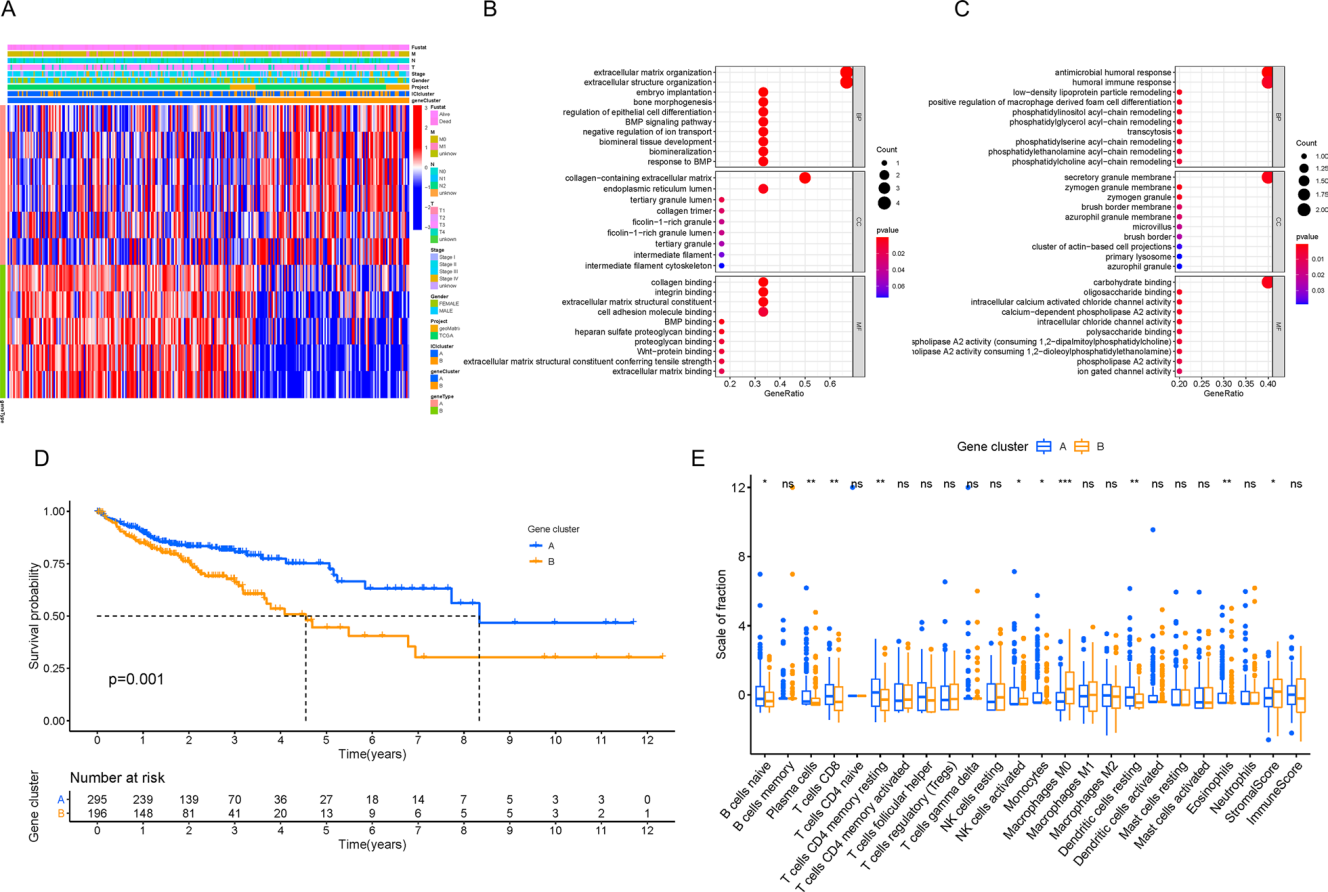
Determination of Immunogenic Gene Subtypes. **A** Unsupervised clustering of DEGs between two ICI clusters to divide patients into two subgroups: gene clusters A–B. GO enrichment analysis of the two ICI-related signature genes: ICI signature genes A (**B**) and B (**C**). **D** Kaplan–Meier plotter for COAD patients from two ICI gene clusters. **E **The subpopulation of tumor infiltrating immune cells, stromal scores and immune scores in ICI gene cluster **A** and **B**

**Fig. 4:**
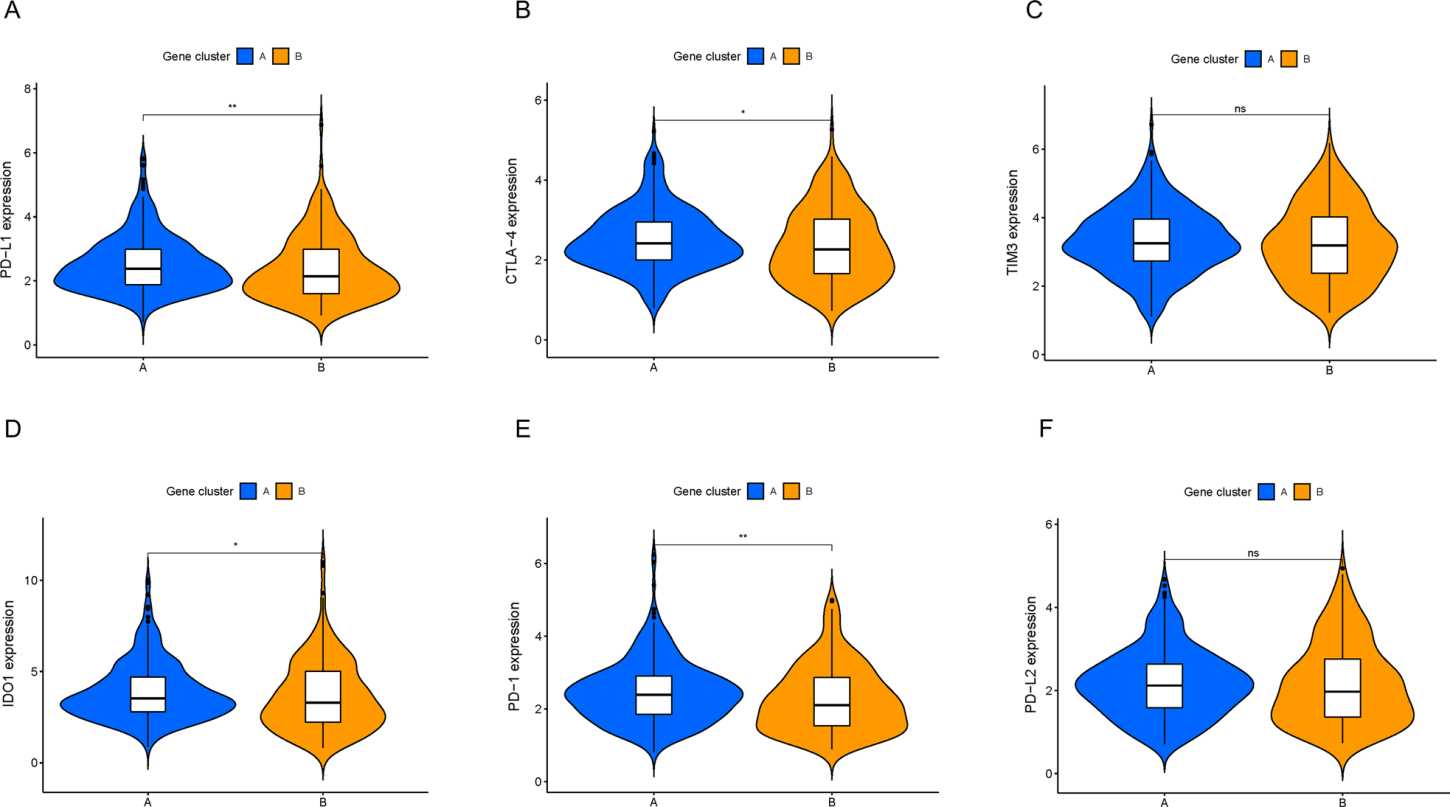
The contrast of ICB genes between two ICI gene clusters. The expression levels of PD-L1 (**A**), CTLA4 (**B**), TIM3 (**C**), IDO1 (**D**), PD1 (**E**) and PD-L2 (**F**) of COAD patients from distinct ICI gene clusters

In order to reveal the potential biological characteristics of different immunophenotypes, we conducted differential gene expression analysis to identify the genomic variations between these two subsets using the R software package “limma.” As described previously, we selected 11 DEGs (Additional file 1: Table S3) and grouped 538 CRC patients from the TCGA-COAD and GSE29623 combination cohort into two transcriptomic subgroups via unsupervised clustering (gene clusters A and B; Additional file 3: Figure S2A–F). Among the 11 DEGs, 6 genes were positively correlated with the gene cluster, which was termed gene signature A, whereas the other 5 genes were classified as gene signature B (Additional file 1: Table S4). In Fig. 3A, we depicted the heatmap to visualize the transcriptomic variation between gene cluster A and gene cluster B using the R software package “ClusterProfiler.” In addition, we performed GSEA analysis to elucidate the significantly enriched biological processes of gene signatures A and B (Fig. 3B and C), and more details are presented in Additional file 1: Table S5. To investigate the prognostic value of immune gene clustering, we performed survival analysis by drawing a Kaplan–Meier curve. We found that patients in cluster A had longer OS than those in gene cluster B, which indicates a better prognosis (Fig. 3D; P = 0.001). Subsequently, we determined the difference in TME characterization between gene cluster A and gene cluster B. As shown in Fig. 3E, gene cluster A exhibited higher infiltration of naïve B cells, plasma cells, CD8 T cells, resting memory CD4 T cells, activated NK cells, monocytes, and eosinophils. However, gene cluster B showed remarkably high expression of M0 macrophages and a higher stromal score. Furthermore, we compared the expression levels of the six aforementioned ICB genes between the two gene clusters. As depicted in Fig. 4A–F, gene cluster A was characterized by upregulated levels of PD-L1, CTLA-4, IDO-1, and PD-1, whereas TIM3 and PD-L2 levels were not significantly different.

### Generation of ICI scores in COAD

**Fig. 5:**
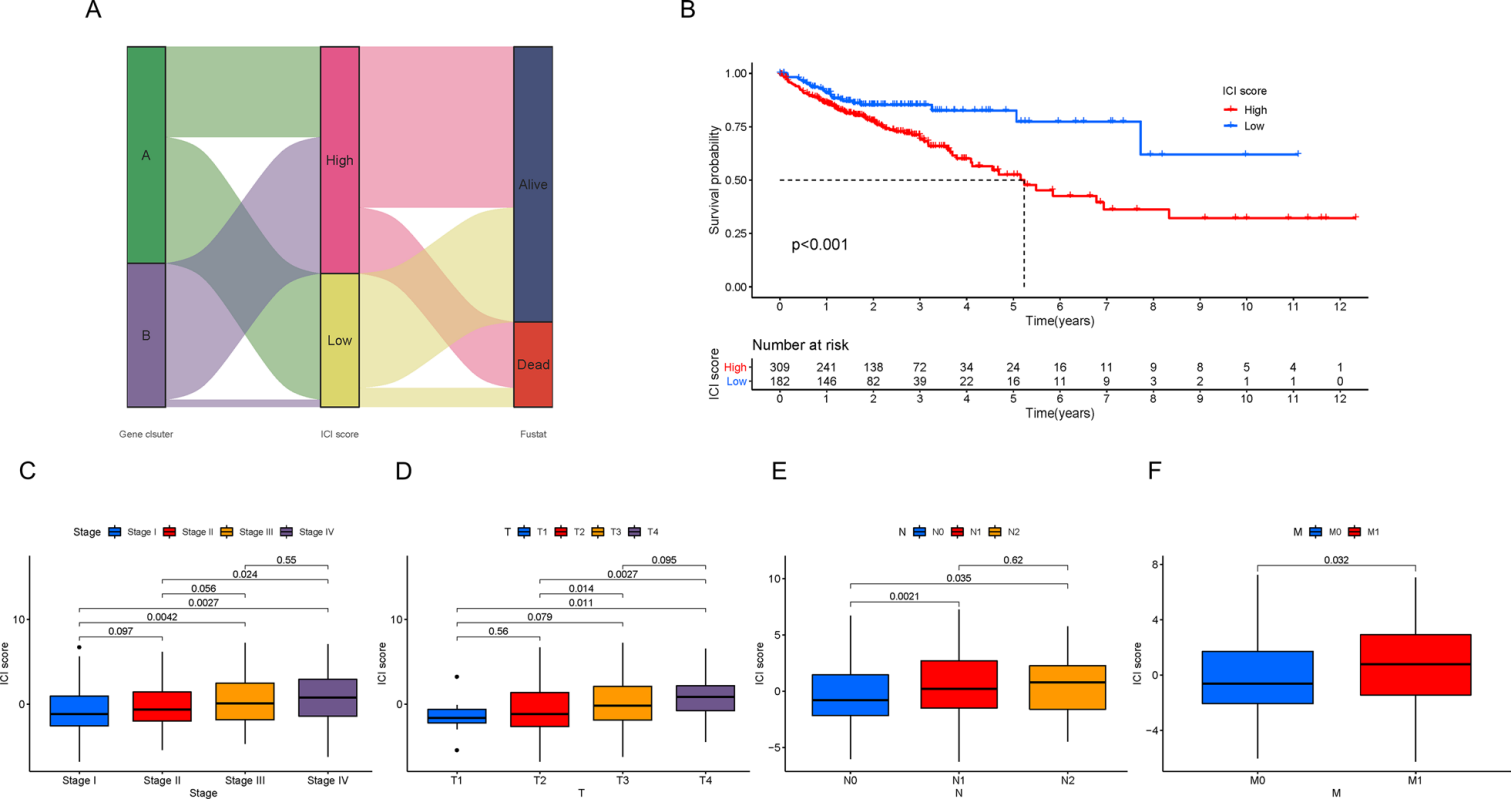
Generation of the ICI Scores. **A** Sankey diagram of the distribution of ICI gene clusters in subgroups with dissimilar ICI scores and survival state. **B** Kaplan–Meier plotter for high and low ICI score subgroups. Comparison of ICI score among different subgroups classified on the basis of clinical characteristics: caner staging (**C**), tumor (**D**), regional lymph node (**E**) and metastasis (**F**)

To quantitatively estimate the ICI profiles of CRC patients, we calculated the two parts of the scores from ICI gene signatures A and B using PCA. Subsequently, we used X-tile software to obtain the best cutoff value and classified all patients from the TCGA-COAD and GSE29623 cohort into two subgroups named “high ICI scores” and “low ICI scores.” The specific distribution is shown in the Sankey diagram (Fig. 5A). Using the Kaplan–Meier plotter, we evaluated the prognostic value of the ICI score and found that patients with low ICI scores had a better survival advantage compared to the high ICI score subset in both the combination and TCGA-COAD cohorts (Fig. 5B; P < 0.01 and Additional file 4: Figure S3A; P < 0.001). Although there was no significant difference, we observed that patients with relatively low ICI scores had a better survival probability in the GSE29623 cohort (Additional file 4: Figure S3B; P = 0.526). Furthermore, we performed stratification analysis and divided the patients from the combination cohort into different subgroups based on clinical characteristics (Additional file 4: Figure S3C–H). In Fig. 5C–F, we found that ICI scores were positively correlated with tumor staging and tumor–node–metastasis (TNM) stage. A higher ICI score often indicated a more advanced tumor and a worse prognosis, which is also consistent with the results of the aforementioned Kaplan–Meier curves. In addition, sex did not appear to be related to ICI score (Additional file 4: Figure S3I). Together, this consistency among clinical characteristics and prognosis confirmed the scientific and rational nature of our classification methods. To further analyze the prognostic predictive ability of the ICI score in different clinical characteristic subgroups, we depicted the survival curves. As shown in Additional file 5: Figure S4A–4 J, we discovered that the ICI score had promising predictive value in patients who were male, T3–4, N0, M0, and stage I–II.

### Relationship between ICI score and immunotherapy

**Fig. 6:**
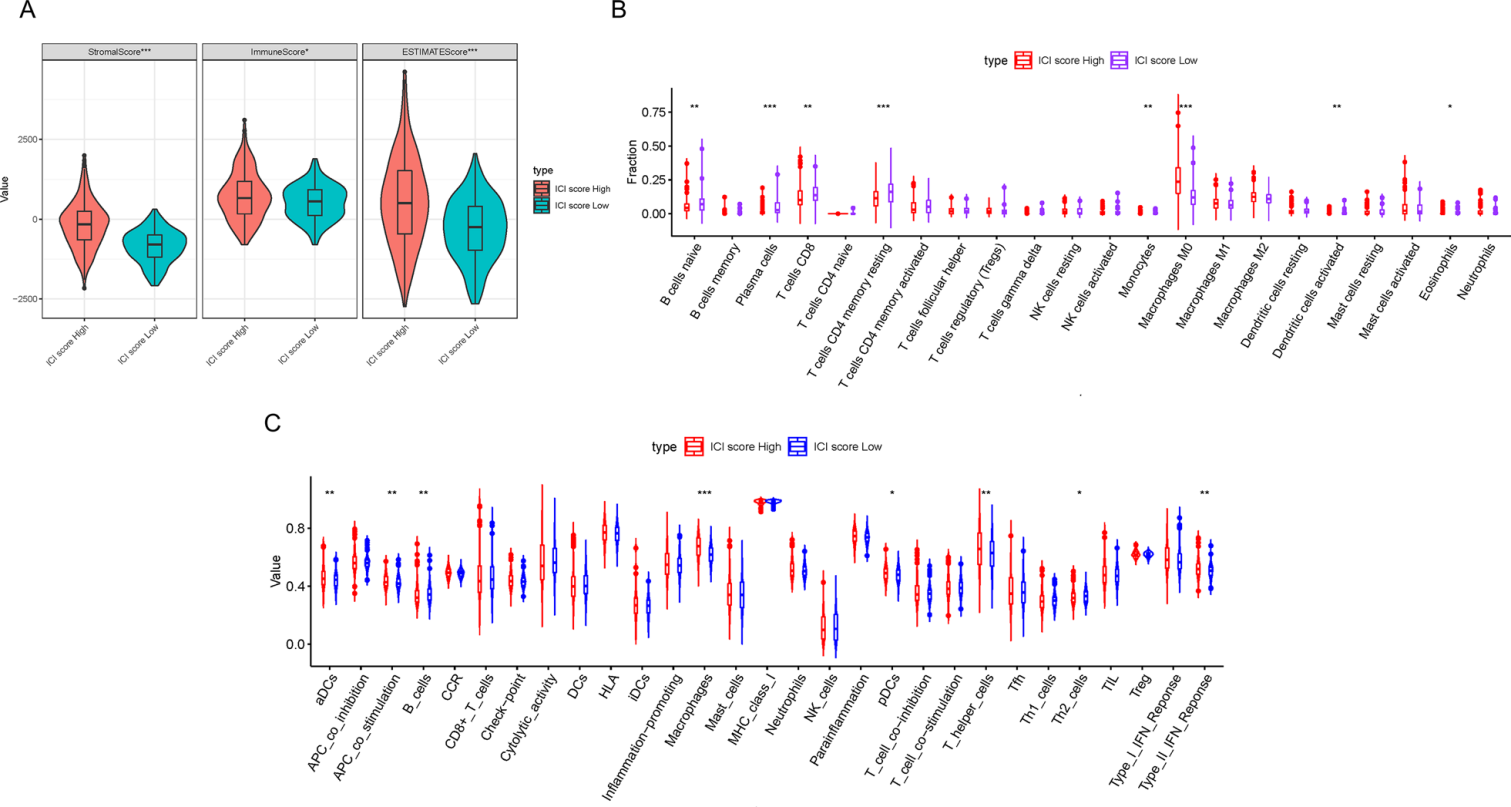
Correlation between ICI scores and the characterization of TME. **A** Comparison of stromal scores, immune scores and estimate scores between patients with high ICI scores and patients with low ICI scores. **B** The fraction of tumor infiltrating immune cells between two ICI score subgroups. **C** The value of 29 immune-related signatures between high and low ICI score subgroups

To further elucidate the connection between ICI score and ICI patterns, we calculated stromal, immune, and estimate scores using the ESTIMATE algorithm. As depicted in Fig. 6A, we found that the high ICI score subgroup had higher stromal, immune, and estimate scores. Subsequently, with the help of the R software package “CIBERSORT,” we uncovered that the fraction of active immune cells, such as naïve B cells, plasma cells, CD8 T cells, resting memory CD4 T cells, monocytes, activated dendritic cells and eosinophils, were negatively correlated with ICI score, whereas M0 macrophages were positively associated with ICI score (Fig. 6B). In addition, we used the ssGSEA algorithm to estimate the immune-related signatures. As shown in Fig. 6C, B cells were relatively high in patients with low ICI scores, but there was an increasing trend of macrophages in the high ICI score subgroup.

**Fig. 7:**
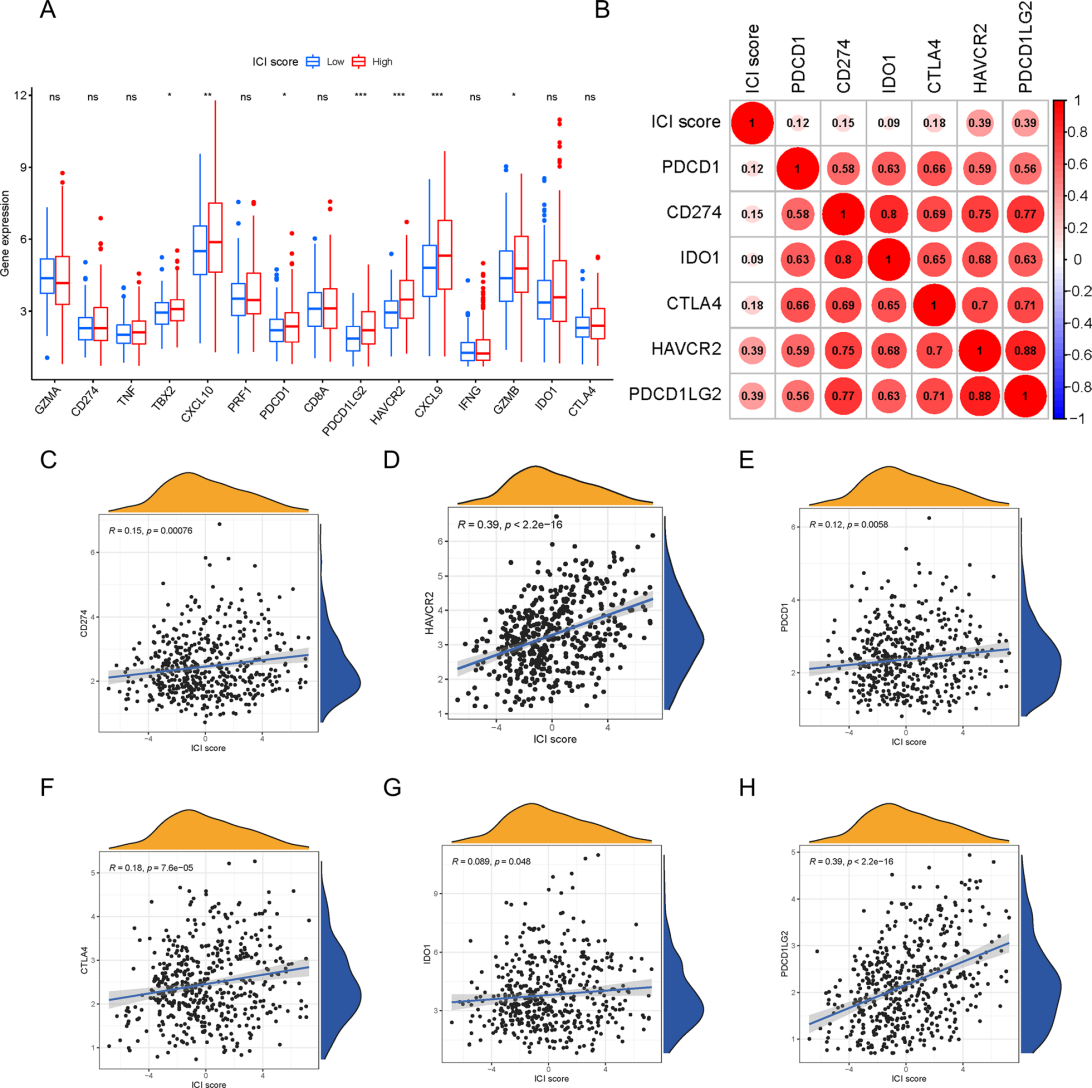
Immunotherapeutic Significance of ICI Scores. **A** The expression level of ICB-related genes (CD274, PDCD1, PDCD1LG2, HAVCR2, IDO1 and CTLA4) and inflammatory-relevant genes (GZMA, TNF, TBX2, CXCL10, PRF1, CD8A, CXCL9, IFNG and GZMB) in high- / low- ICI score subgroups. **B** Correlation analysis between ICI scores with crucial immune checkpoint inhibitors (PDCD1, CD274, IDO1, CTLA4, HAVCR2 and PDCD1LG2). Correlation between ICI scores and ICB key target genes, including CD274 (**C**), HAVCR2 (**D**), PDCD1 (**E**), CTLA4 (**F**), IDO1 (**G**) and PDCD1LG2 (**H**)

To assess the immune activity or tolerance condition of the two ICI score subgroups, we presented the expression level profiles of six ICB genes (CD274, CTLA4, HAVCR2, IDO1, PDCD1, and PDCD1LG2) and nine inflammation-related genes (CD8A, CXCL10, CXCL9, GZMA, GZMB, IFNG, PRF1, TBX2, and TNF). As shown in Fig. 7A, we found that several genes, particularly HAVCR2, PCDC1LG2, CXCL9, and CXCL10, were highly expressed in patients with high ICI scores. Subsequently, we further estimated the relationship between ICI score and ICB key target genes and discovered that all six ICB genes were positively correlated with ICI score (Fig. 7B–H; CD274 [R = 0.15, P = 0.00076], CTLA4 [R = 0.18, P = 7.6e-05], HAVCR2 [R = 0.39, P < 2.2e − 16], IDO1 [R = 0.089, P = 0.048], PDCD1 [R = 0.12, P = 0.0058], and PDCD1LG2 [R = 0.39, P < 2.2e − 16]).

### Correlation between ICI score and tumor mutational burden (TMB)

**Fig. 8:**
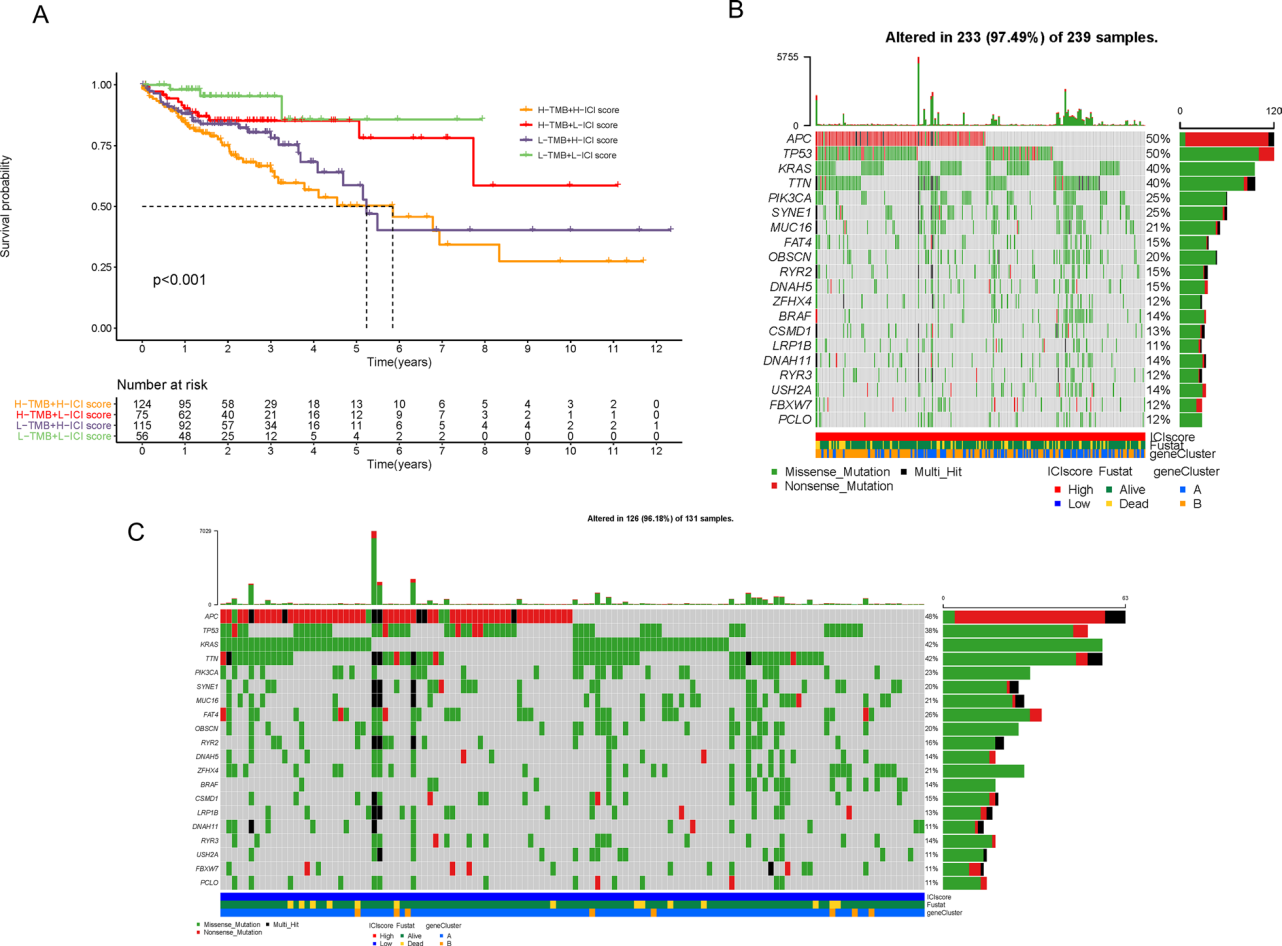
The Correlation of ICI Scores with TMB. **A** Kaplan–Meier plotter for patients stratified by both ICI scores and TMB. The oncoPrint was constructed with high ICI scores (**B**) and low ICI scores (**C**)

Recently, many original studies have elucidated that high TMB may be utilized as a new biomarker to predict the effect of immunotherapy [15]. Considering this newfound clinical significance of TMB, we explored the link between TMB and ICI score. First, we compared the TMB value between the high and low ICI score subgroups and found no significant difference (Additional file 6: Figure S5A). The results of correlation analysis revealed that there was no obvious correlation between TMB and ICI score (Additional file 6: Figure S5B; R = − 0.1, P = 0.052). Subsequently, we conducted survival analysis and demonstrated that the OS of patients with high or low TMB was not statistically different (Additional file 6: Figure S5C; P = 0.113). In order to further verify the prognostic value of the ICI score and exclude the interference of TMB, we performed stratified survival analysis and found that regardless of the TMB level, patients with low ICI scores exhibited a more optimal survival advantage compared to patients with high ICI scores (Fig. 8A; P < 0.01). In addition, we evaluated the distribution of COAD gene mutations in the low and high ICI score subgroups. As shown in Fig. 8B and C, we drew the comprehensive patterns of somatic variants and listed the top 20 driver genes with the highest alternative frequencies. Taken together, these results illustrate that ICI score may act as a potential independent prognostic indicator and be used to guide ICB therapy.

### The prognostic roles of ITLN1 and CLCA1

**Fig. 9:**
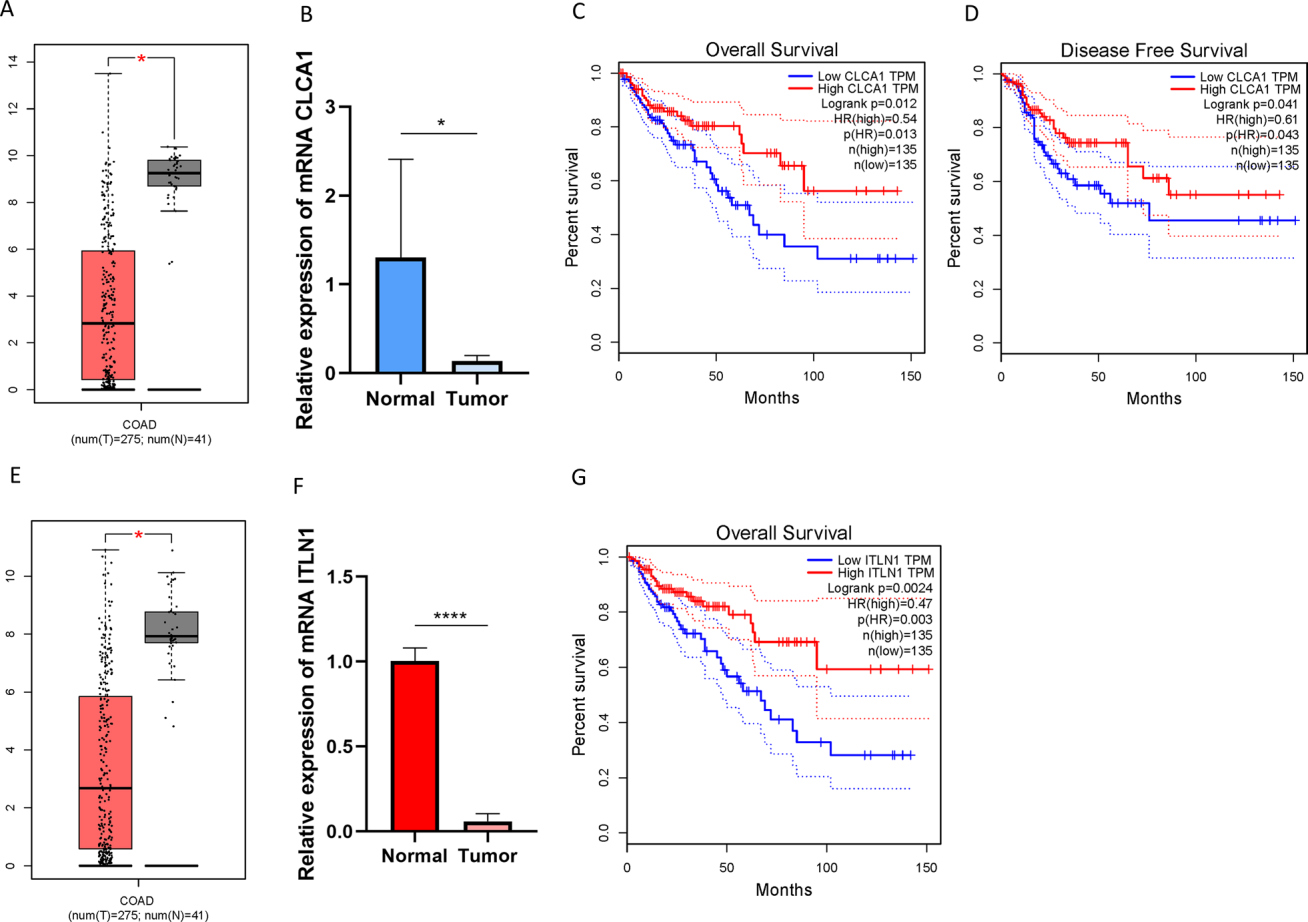
The clinical significance of CLCA1 and ITLN1. CLCA1 are downregulated in COAD tumor tissue based on TCGA dataset (**A**) and experimental study (**B**). Higher CLCA1 level predicts longer overall survival time (**C**) and disease-free survival time (**D**). ITLN1 are donwrefulated in COAD tumor tissue based on TCGA dataset (**E**) and experimental study (**F**). Higher CLCA1 level predicts longer overall survival time (**G**)

To better reveal the prognostic role of potential targets from ICI gene signatures A and B, two genes (ITLN1 and CLCA1) were extracted into further analyses. Chloride channel accessory 1 (CLCA1), one of CLCA family protein, served as a functional player in modulating the proliferation and differentiation [16]. The expression value of CLCA1 has not yet been investigated from the mRNA level in COAD although Bo Yang et al. reported that CLCA1 could be a predictor of prognosis in primary human CRC. Expression level of CLCA1 between normal tissues and tumor samples was compared based on TCGA data. Relative to tumor tissues, CLCA1 was upregulated in adjacent normal specimens (Fig. 9A). Taking advantage of qRT-PCR, expression level of CLCA1 in COAD tissues and adjacent samples were determined. Consistent of previous results, expression level of CLCA1 was lower in tumor relative to adjacent tissues (Fig. 9B). To further estimate the prognostic value of CLCA1 in COAD, Kaplan–Meier analysis were conducted between CLCA1 low- and high-expressed patients. As presented in Figs. 9C and D, low expression level of CLCA1 significantly suggested shorter overall survival time (P = 0.012) and longer disease-free survival time (P = 0.041). These findings indicated that CLCA1 could be a robust prognostic factor for predicting clinical outcome in COAD.

Human intelectin-1 (ITLN1) is a novel recognized galactose-binding lectin expressed in the colonic goblet cells. The aberrant ITLN1 expression has been demonstrated to correlate with clinicopathological features and be a robust predicting indicator for prognosis of gastric cancer patients [17]. However, the prognostic role of ITLN1 in COAD remains unclear. Our results showed that expression level of ITLN1 was downregulated in cancer compared with normal tissues based on TCGA dataset (Fig. 9E) and experimental validation (Fig. 9F). As for survival analysis, high expression level of ITLN1 experienced significant prognosis advantage (Fig. 9F, P= 0.0024). Our results indicated that ITLN1 might serve as a reliable biomarker for prognostic prediction in COAD.

## Discussion

CRC is the third most common cancer, accounting for 9% of cancer-related deaths in both men and women [1]. Owing to high intertumor and intratumor heterogeneity, different patients, even those with the same TNM stage, have different survival rates [18]. Surgery, radiotherapy, and chemotherapy are the main treatments for CRC. Recently, immunotherapy has been regarded as a promising therapeutic method for CRC. Two immune checkpoint inhibitors, pembrolizumab and nivolumab, have been approved by the U.S. Food and Drug Administration and verified to be effective in metastatic CRC patients with high microsatellite instability (MSI-H) and dMMR [19, 20]. However, only approximately 5% of patients with metastatic CRC (complying with dMMR/MSI-H) can benefit from immunotherapy, and many immune-sensitive patients can be distinguished via molecular subtyping, signature of gene expression, or immune-related score [21]. Thus, there is an urgent need to identify an original and effective biomarker for predicting prognosis and guiding immunotherapy options.

The characteristics of inflammatory infiltration indicate that there are significant differences in the number and location of inflammatory cells in different colorectal tumor types. For example, patients with dMMR/MSI-H often possess more tumor-infiltrating lymphocytes and a higher TMB, which reduces immune tolerance and immune evasion in the TME. In this study, we estimated the ICI patterns of 538 COAD samples from the TCGA-COAD and GSE29623 combination cohorts and divided them into two different ICI clusters using consensus clustering. Our results showed that ICI cluster A had significantly greater proportions of plasma cells, CD8 T cells, CD4 T cells, dendritic cells, activated NK cells, and M1 macrophages as well as a higher immune score, whereas ICI cluster B possessed markedly increasing densities of regulatory T cells and M0 macrophages as well as a higher stromal score. It appeared that patients in ICI cluster A may have a pre-existing anti-tumor immune response compared to patients in ICI cluster B. However, the survival analysis results between the two clusters were not significantly different. This illustrates that immune phenotypes cannot accurately evaluate the prognosis of patients with COAD and respond to immunotherapy.

A recent study revealed that molecular subtype-specific biomarkers have high prognostic value in CRC [22]. Therefore, we integrated the characterization of ICI patterns and immune-related gene expression profiles to provide a more original and comprehensive classification scheme. In the current study, we defined six DEGs as gene signature A, which positively correlated with the gene cluster, and the remaining DEGs were termed gene signature B. Our data showed that ICI gene cluster A contained more genes from signature B, which was associated with the immune response, whereas ICI gene cluster B displayed higher expression levels of gene signature A, which was related to the extracellular matrix and structure organization. In line with these results, patients in ICI gene cluster A exhibited higher densities of plasma cells and CD8 T cells in the TME, but patients in ICI gene cluster B had a relatively high stromal score, which was likely also related to higher tumor-associated macrophage cell infiltration. Survival analysis revealed that ICI gene cluster A had a more favorable prognosis than ICI gene cluster B based on the Kaplan–Meier plotter. In addition, ICI gene cluster A was characterized by upregulated levels of four ICB genes: PD-L1, CTLA-4, IDO-1, and PD-1. Consequently, we speculated that patients in ICI gene cluster A may be more suitable for immune-related therapy.

Given the complex tumor heterogeneity, we quantified the ICI profiles of each patient. In this study, based on the Boruta algorithm, we constructed an ICI score to comprehensively present the ICI landscape. Our results showed that ICI scores were positively correlated with tumor staging and TNM stage. A higher ICI score often indicates a more advanced tumor and worse prognosis. With the help of the ESTIMATE, CIBERSORT, and ssGSEA algorithms, we observed that the high ICI score subgroup had a higher stromal score and more M0 macrophage infiltration. Although the high ICI score subset had a higher immune score, there was a clearly higher distribution of plasma cells and CD8 T cells in the TME of the low ICI score subgroup. However, interestingly, patients with high ICI scores showed significantly higher expression levels of HAVCR2 (also named TIM3) and PCDC1LG2 (also named PD-L2). At present, immunotherapy for CRC mainly focuses on PD1/PD-L1, and two drugs, pembrolizumab and nivolumab, have been approved for use in metastatic CRC patients. As the research develops, TIM3 and PD-L2 may become new targets for COAD immunotherapy, bringing clinical benefits to patients with advanced tumors in the high ICI score subgroup. Finally, in order to exclude the interference of TMB, we performed stratified survival analysis and discovered that the ICI score may independently act as a potential prognostic indicator that can analyze the response to immunotherapy.

Chloride channel accessory 1 (CLCA1) as a member of CLCA family protein, functioned as a biological role in modulating the proliferation and differentiation [16]. Furthermore, CLCA1 might serve as opposing role in progression of tumor [23]. Human ITLN1 is produced from the goblet cells and secreted into mucus in normal colon epithelia [24], and expression level of ITLN1 was upregulated during gastrointestinal infection [25]. However, little to know about the prognostic role of both CLCA1 and ITLN1 in COAD. The present study shows that CLCA1 and ITLN1 is downregulated in COAD tissues, could function as favorable prognostic predictors in COAD. However, the underlying biological mechanism requires to be further investigated in future experiment.

In conclusion, we comprehensively evaluated and quantified the ICI patterns of COAD, clarified that patients with different ICI patterns have different prognoses and clinical outcomes, and provided potential targets for COAD immunotherapy. Therefore, systematic analysis of ICI patterns may be considered as an original and useful indicator for prognostic prediction and individual immune-related therapy.

## Supplementary Information

**Additional file 1.** Additional tables.

**Additional file 2: Figure S1.** (A-D) Consensus matrixes of COAD patients for each k (from 2 to 5), and the clustering stability was displayed by using 1000 hierarchical clustering iterations. (E) The relative changes in area under the CDF curve for index k (from 2 to 9). (F) Area under CDF curves when k ranges from 2 to 9.

**Additional file 3: Figure S2.** (A-D) Consensus matrixes of COAD patients for each k (from 2 to 5), and the clustering stability was displayed via employing 1000 hierarchical clustering iterations. (E) The relative changes in area under the CDF curve for index k (from 2 to 9). (F) Area under CDF curves when k ranges from 2 to 9.

**Additional file 4: Figure S3.** (A-B) Kaplan-Meier plotters for high-/low- ICI score groups in the TCGA-COAD database (A) and GSE29623 cohort (B). (C-H) The proportion of COAD patients stratified by different clinical characteristics in high and low ICI score subgroups. (I) Comparison of ICI score between male and female subgroups.

**Additional file 5: Figure S4**. Kaplan-Meier plotters for patients stratified by various clinical characteristics, such as gender (A-B), metastasis (C-D), regional lymph node (E-F), caner staging (G-H), and tumor (I-J).

**Additional file 6: Figure S5. **(A) Difference of TMB between high-/low- ICI score subgroups. (B) Scatterplots depicting no significant correlation between ICI scores and TMB in TCGA-COAD cohort. (C) Kaplan-Meier plotters for patients with high and low TMB.

## Data Availability

The study was based on the data available at TCGA (https://www.cancer.gov/tcga) and GEO (https://www.ncbi.nlm.nih.gov/geo/).
