# Supplementary figures and images for: Prognostic value of tumor immune cell infiltration patterns in colon adenocarcinoma based on systematic bioinformatics analysis
Source: Cancer Cell Int. 2021 Jul 3;21:344. doi: 10.1186/s12935-021-02048-x (PMC8254898; doi:10.1186/s12935-021-02048-x)

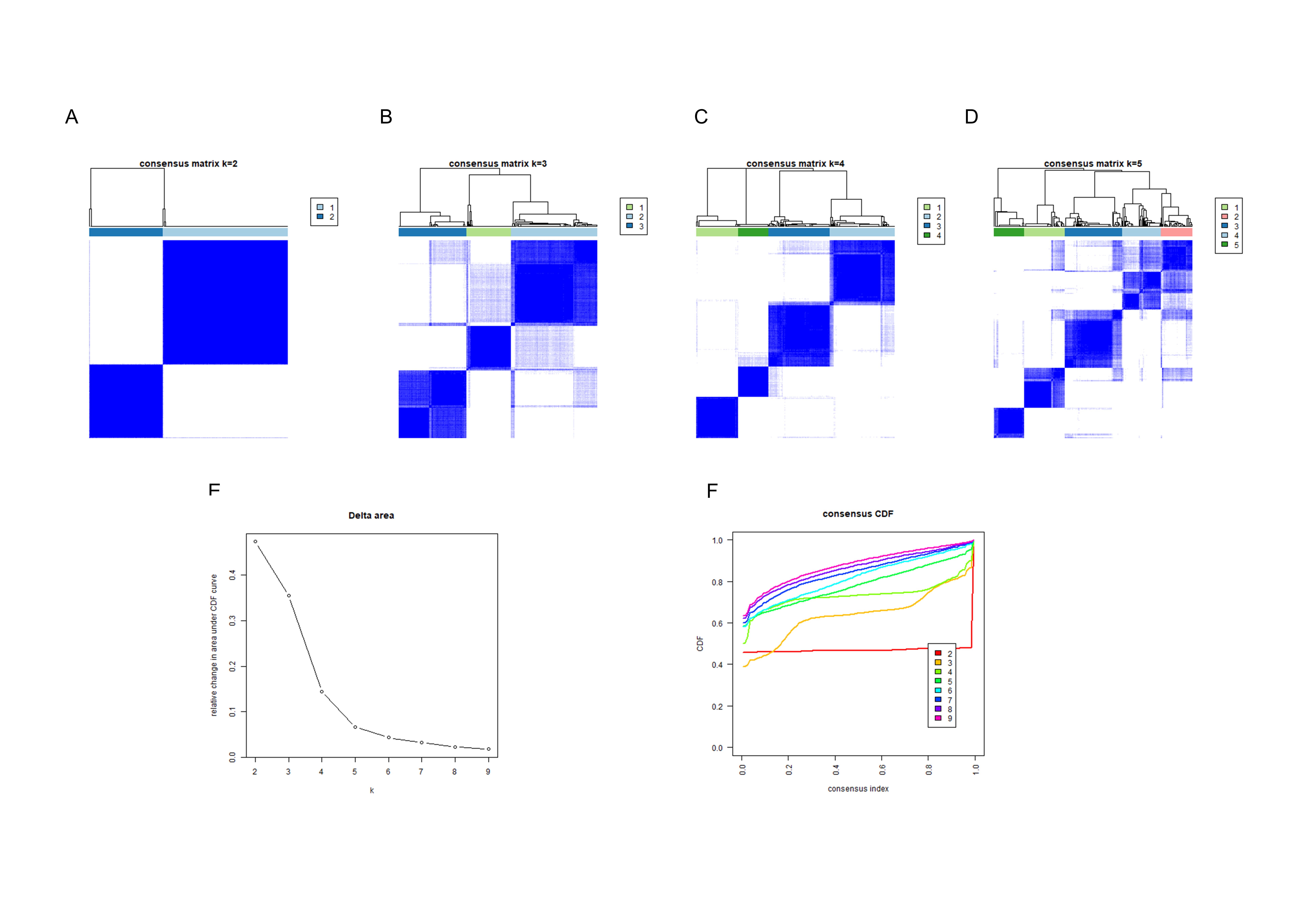

Supplement: Supplementary file 2 — Additional file 2: Figure S1. (A-D) Consensus matrixes of COAD patients for each k (from 2 to 5), and the clustering stability was displayed by using 1000 hierarchical clustering iterations. (E) The relative changes in area under the CDF curve for index k (from 2 to 9). (F) Area under CDF curves when k ranges from 2 to 9. [file 12935_2021_2048_MOESM2_ESM.tif]

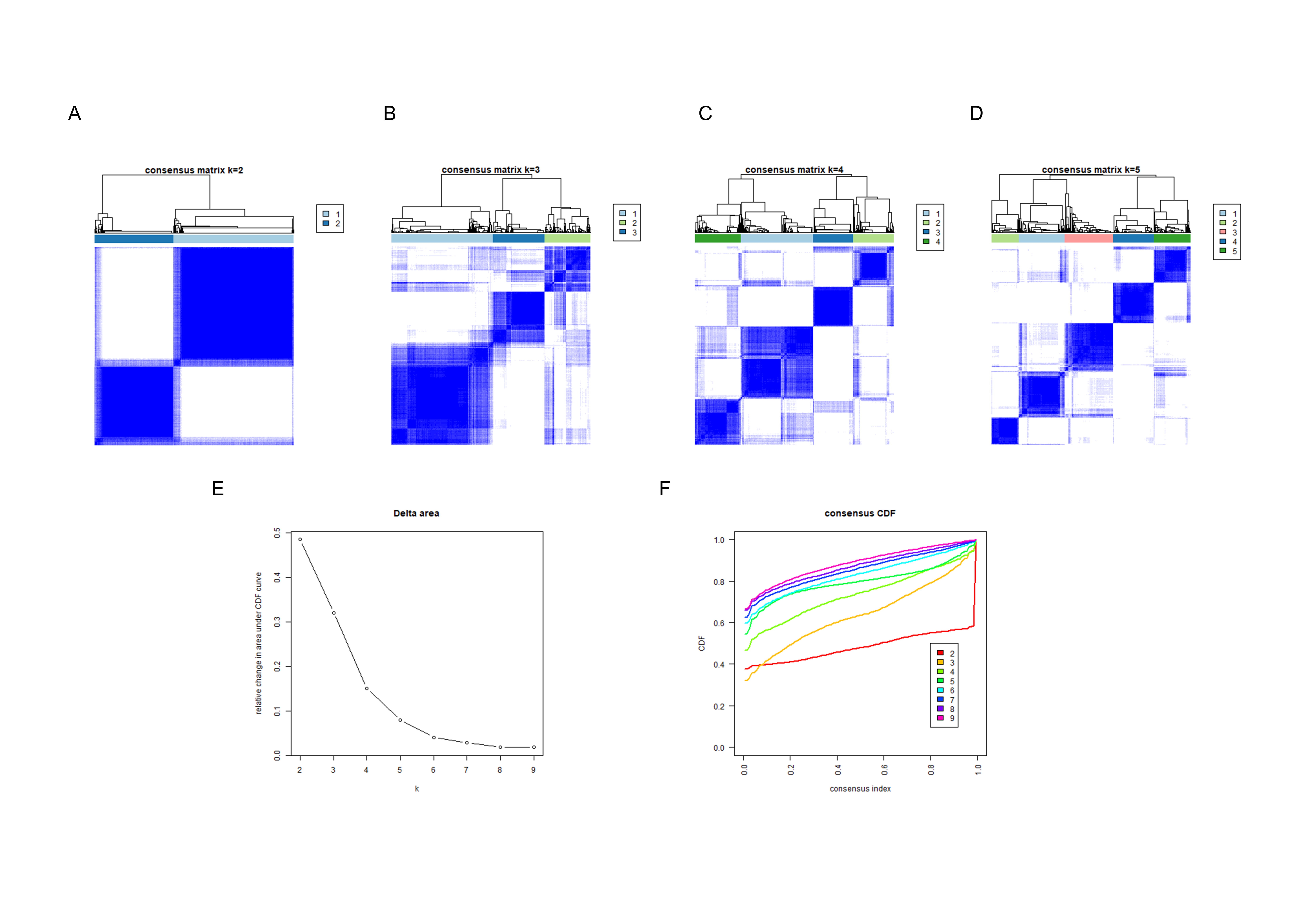

Supplement: Supplementary file 3 — Additional file 3: Figure S2. (A-D) Consensus matrixes of COAD patients for each k (from 2 to 5), and the clustering stability was displayed via employing 1000 hierarchical clustering iterations. (E) The relative changes in area under the CDF curve for index k (from 2 to 9). (F) Area under CDF curves when k ranges from 2 to 9. [file 12935_2021_2048_MOESM3_ESM.tif]

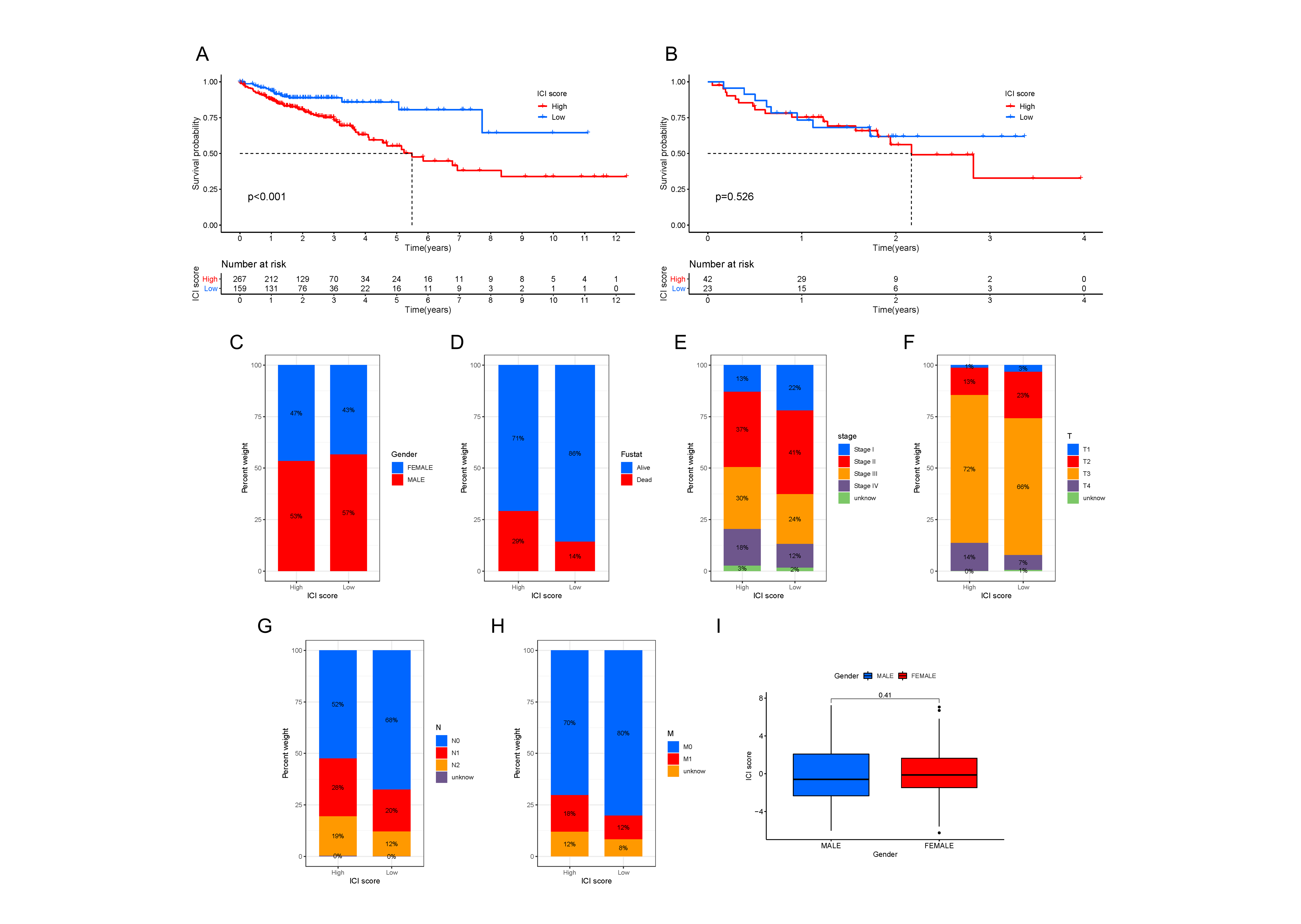

Supplement: Supplementary file 4 — Additional file 4: Figure S3. (A-B) Kaplan-Meier plotters for high-/low- ICI score groups in the TCGA-COAD database (A) and GSE29623 cohort (B). (C-H) The proportion of COAD patients stratified by different clinical characteristics in high and low ICI score subgroups. (I) Comparison of ICI score between male and female subgroups. [file 12935_2021_2048_MOESM4_ESM.tif]

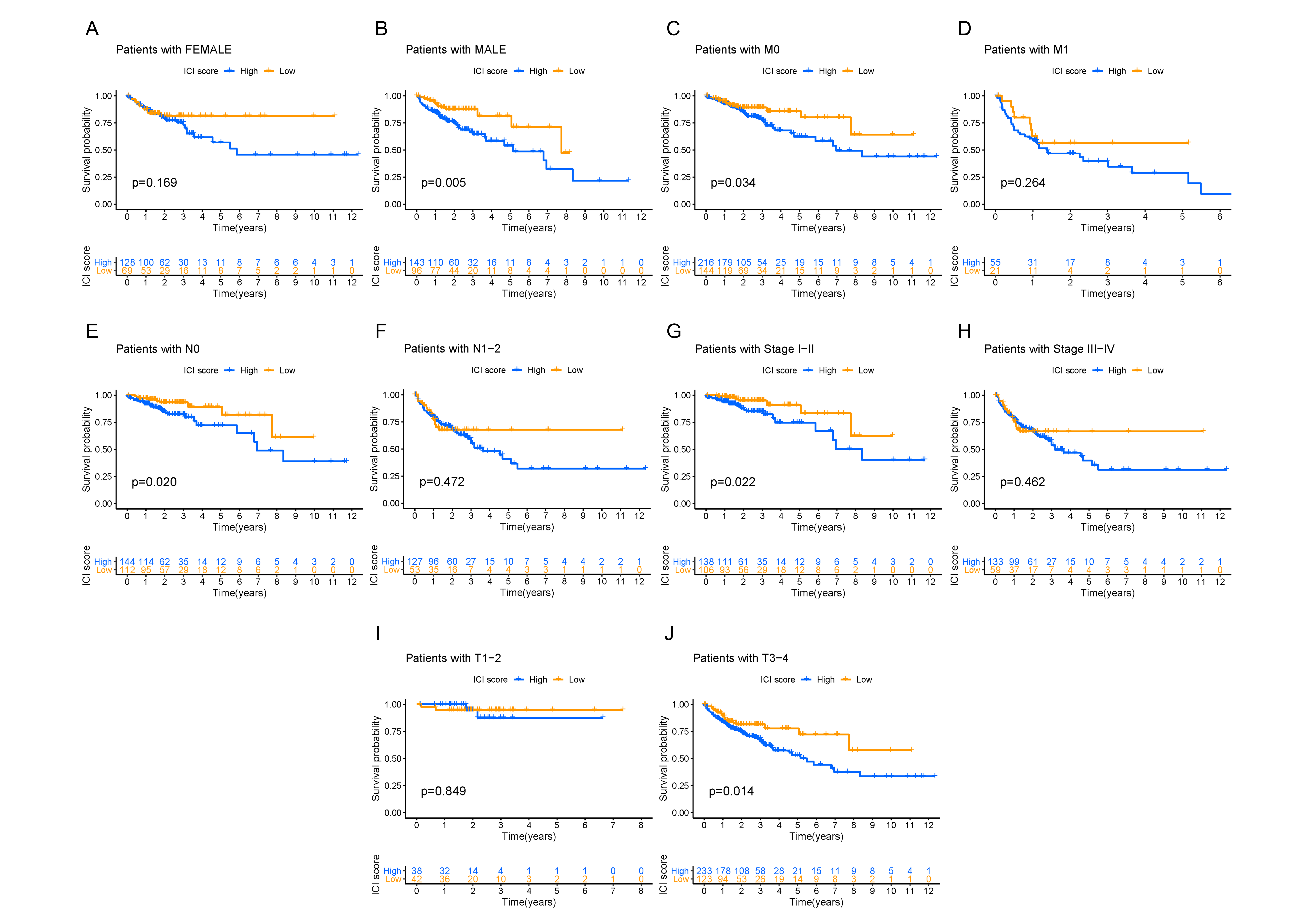

Supplement: Supplementary file 5 — Additional file 5: Figure S4. Kaplan-Meier plotters for patients stratified by various clinical characteristics, such as gender (A-B), metastasis (C-D), regional lymph node (E-F), caner staging (G-H), and tumor (I-J). [file 12935_2021_2048_MOESM5_ESM.tif]

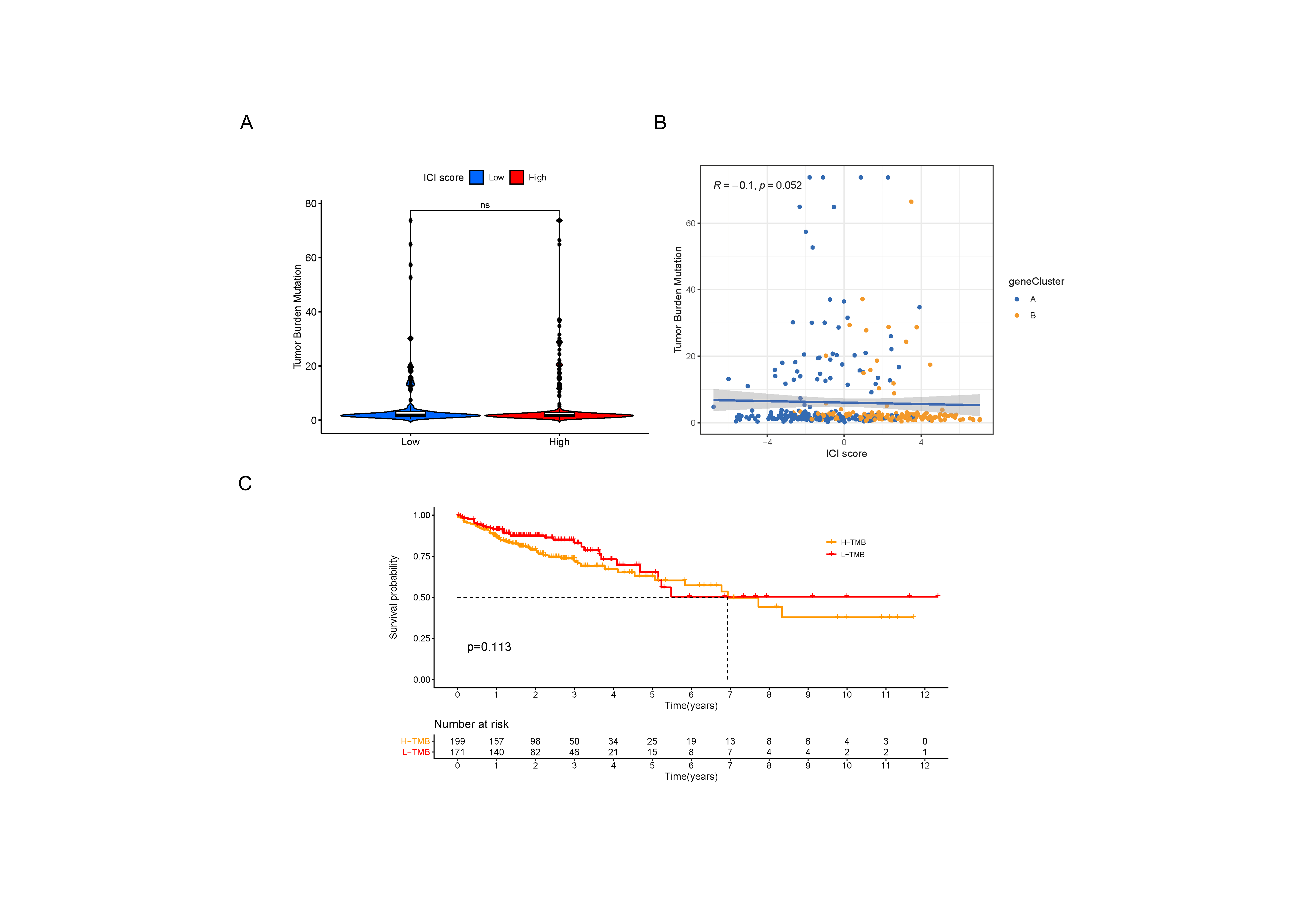

Supplement: Supplementary file 6 — Additional file 6: Figure S5. (A) Difference of TMB between high-/low- ICI score subgroups. (B) Scatterplots depicting no significant correlation between ICI scores and TMB in TCGA-COAD cohort. (C) Kaplan-Meier plotters for patients with high and low TMB. [file 12935_2021_2048_MOESM6_ESM.tif]
